# Multiple Prostatic Abscesses Caused by Staphylococcus aureus Without Physical Findings in an Immunosuppressed Older Patient

**DOI:** 10.7759/cureus.33555

**Published:** 2023-01-09

**Authors:** Junji Uchiyama, Yudai Tanaka, Yasuo Kurita, Chiaki Sano, Ryuichi Ohta

**Affiliations:** 1 Family Medicine, International University of Health and Welfare, Tokyo, JPN; 2 Community Care, Unnan City Hospital, Unnan, JPN; 3 Cardiology, International University of Health and Welfare, Tokyo, JPN; 4 Community Medicine Management, Shimane University Faculty of Medicine, Izumo, JPN

**Keywords:** antibacterial drug, gangrenous cholecystitis, diabetes mellitus, prostatic abscess, staphylococcus aureus bacteremia, rural hospital, general medicine

## Abstract

*Staphylococcus aureus* is endemic to human and animal skin and the gastrointestinal tract and is highly tissue-destructive. *Staphylococcus aureus* bacteremia has a high mortality rate of 20%-30%. A prostatic abscess is a rare complication of acute bacterial prostatitis. The focus of *S. aureus* infection is elsewhere in the body, and bacteremia causes the abscess, hence difficult to diagnose. Here, we report a case of prostatic abscesses, followed by a diagnosis of *S. aureus *bacteremia without specific physical findings. The patient was a 72-year-old male with independent activities of daily living who developed prostate and perifemoral abscesses with multiple vague symptoms due to diabetes-related methicillin-susceptible *S. aureus* bacteremia. It is important to comprehensively evaluate multiple vague symptoms considering the immunological conditions of patients and investigate any suspicion of bacteremia and abscess in deep parts of the body. General physicians should be system-specific specialists to deal with multiple symptoms among older immunocompromised patients.

## Introduction

*Staphylococcus aureus* is endemic to human and animal skin and the gastrointestinal tract and is highly tissue-destructive. *Staphylococcus aureus* bacteremia has a high mortality rate of 20%-30% [1] and a poor prognosis if not treated appropriately. The probability of contamination of blood cultures with *S. aureus* is extremely low, and even one positive blood culture should be treated as *S. aureus* bacteremia. *Staphylococcus aureus* bacteremia carries a high risk for various complications, including prostate abscesses, osteomyelitis, and infectious endocarditis [2].

Prostatic abscesses caused by *S. aureus* can be difficult to diagnose and treat in immunocompromised hosts [2]. Usually, prostate abscesses show several symptoms such as spiking fever, malaise, irritative urinary symptoms, pelvic or perineal pain, and/or cloudy urine. Rectal examination usually reveals tenderness and rippling of the prostate, which is a localized accumulation of pus that occurs as a complication of acute bacterial prostatitis and other tract infections. However, immunocompromised conditions can mask physical findings. Here, we report a case of prostatic abscess diagnosed following the diagnosis of *S. aureus* bacteremia. In this case, we discuss the difficulties in diagnosing the disease and practical methods in rural hospitals.

## Case presentation

A 72-year-old male presented to our hospital with chief complaints of anorexia and dysuria. The patient had experienced anorexia two weeks before the visit. Three days before the visit, he had mild pain during urination, but the pain disappeared at the visit. The patient did not feel chill or abdominal and pelvic pain. The patient did not have sexual partners and did not have any sexual contact within six months. The patient had no history of hospital visits or medical history. Vital signs on admission were as follows: clear consciousness, body temperature of 36.5°C, blood pressure of 116/74 mmHg, heart rate of 114 beats/minute, respiratory rate of 20 breaths/minute, and 100% SpO2 (room air). Physical examination revealed no abnormalities in the head, chest, or abdomen. The physical examination clarified the tenderness at the proximal interphalangeal joints of both hands’ second and third fingers and mild tenderness at the metacarpophalangeal joints and shoulder joints without any joint swelling. The digital rectal examination did not reveal any tenderness of the prostate.

Blood tests revealed an elevated neutrophil-predominant white blood cell count (46,300/μL) and C-reactive protein (CRP) (34.75 mg/dL), and blood culture was performed because of the possibility of bacterial infection (Table 1).

**Table 1:** Initial laboratory data of the patient PT, prothrombin time; INR, international normalized ratio; APTT, activated partial thromboplastin time; eGFR, estimated glomerular filtration rate; CK, creatine kinase; CRP, C-reactive protein; SARS-CoV-2, severe acute respiratory syndrome coronavirus 2

Maker	Level	Reference
White blood cells	46.3 × 10^3^/μL	3.5-9.1 × 10^3^/μL
Neutrophils	94.6%	44%-72%
Lymphocytes	1.7%	18%-59%
Monocytes	3.4%	0%-12%
Eosinophils	0%	0%-10%
Basophils	0.3%	0%-3%
Red blood cells	4.54 × 10^6^/μL	3.76-5.50 × 10^6^/μL
Hemoglobin	13.7 g/dL	11.3-15.2 g/dL
Hematocrit	40.6%	33.4%-44.9%
Mean corpuscular volume	89.5 fl	79-100 fl
Platelets	43.8 × 10^4^/μL	13-36.9 × 10^4^/μL
Total protein	7.9 g/dL	6.5-8.3 g/dL
Albumin	2.8 g/dL	3.8-5.3 g/dL
Total bilirubin	2.1 mg/dL	0.2-1.2 mg/dL
Aspartate aminotransferase	45 IU/L	8-38 IU/L
Alanine aminotransferase	28 IU/L	4-43 IU/L
γ-Glutamyl transpeptidase	25 IU/L	<48 IU/L
Lactate dehydrogenase	272 U/L	121-245 U/L
Uric acid	9 mg/dL	3-6.9 mg/dL
Blood urea nitrogen	52 mg/dL	8-20 mg/dL
Creatinine	1.12 mg/dL	0.40-1.10 mg/dL
eGFR	50.2 mL/minute/L	>60.0 mL/minute/L
Serum sodium	123 mEq/L	135-150 mEq/L
Serum potassium	5.2 mEq/L	3.5-5.3 mEq/L
Serum Cl	82 mEq/L	98-110 mEq/L
Serum Ca	9.0 mg/dL	8.8-10.2 mg/dL
Serum phosphorus	4.4 mg/dL	2.7-4.6 mg/dL
Serum magnesium	2.4 mg/dL	1.8-2.3 mg/dL
Serum amylase	24 IU/L	44-132 IU/L
Serum glucose	459 mg/dL	70-110 mg/dL
Hemoglobin A1c	10.4%	5%-6.2%
CK	913 U/L	56-244 U/L
CRP	34.75 mg/dL	<0.30 mg/dL
Blood sedimentation	70 mm	2-10 mm
PT	52.1%	70%-130%
PT-INR	1.40	
APTT	47.3 seconds	25-40 seconds
Fibrinogen degradation products	6.4 μg/mL	<5 μg/mL
SARS-CoV-2	Negative	Negative
Urine test		
Leukocyte	(3+)	(-)
Nitrite	(-)	(-)
Protein	(1+)	(-)
Glucose	(4+)	(-)
Urobilinogen	(-)	(-)
Bilirubin	(-)	(-)
Ketone	(1+)	(-)
Blood	(3+)	(-)

**Figure 1:**
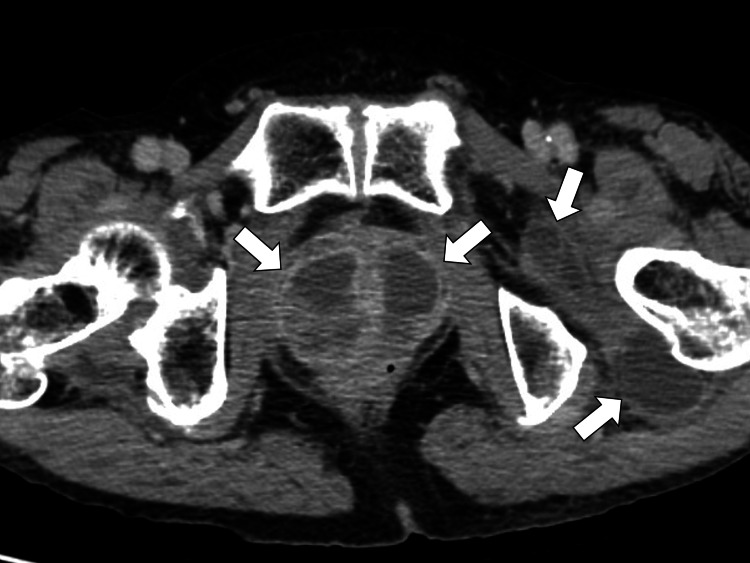
Initial contrast-enhanced computed tomography showing multiple abscess formation in the prostate and perifemoral spaces (white arrows)

Contrast-enhanced computed tomography (CT) scan of the abdomen and pelvis was performed to investigate abscess formation of deep parts of the body, revealing prostate and perifemoral abscesses (Figure 1).

An abscess puncture was performed for the left perifemoral abscess to collect the sample, which showed pale yellow clear viscous drainage and clusters of Gram-positive coccus on Gram staining. Laboratory data also showed hyperglycemia (459 mg/dL), indicating diabetes mellitus. On the second day of hospitalization, the blood test showed a hemoglobin A1c (HbA1c) level of 10.4%, and the blood glucose level remained high at 300-400 mg/dL. We thus treated the patient with insulin lispro and insulin glargine for hyperglycemia. Intravenous vancomycin (1,000 mg) every 24 hours was started for possible *S. aureus* infection.

On day 2 of hospitalization, the patient developed a fever of 38°C, and blood culture on admission showed clusters of Gram-positive coccus, which led to the diagnosis of methicillin-susceptible *S. aureus* (MSSA) bacteremia. Although it was not known at the time of the hospital visit, the patient had fallen while farming a week before the visit and sustained an abrasion on his left thigh. On the third day of hospitalization, a urine culture clarified the susceptibility to MSSA. The antibiotic was changed from vancomycin to cefazolin (2 g every six hours). On day 3 of admission, a second blood culture showed clusters of Gram-positive coccus, again showing persistent blood infection. Sulfamethoxazole 1,600 mg and trimethoprim 320 mg (ST mixture) were administered on day 4 of hospitalization. The fever resolved on day 6 of hospitalization. A third blood culture revealed MSSA with the same antibiotic susceptibility on day 7 after admission.

On the ninth day of hospitalization, his fever increased to over 38°C, accompanied by vomiting and right upper abdominal tenderness. Bedside abdominal ultrasonography revealed an enlarged gallbladder. A simple abdominal CT scan suggested acute cholecystitis. The patient was treated with emergency laparoscopic cholecystectomy. On day 13 of hospitalization, owing to a residual prostatic abscess, drainage was performed under transrectal ultrasonography with approximately 10 mL of greenish-yellow viscous drainage fluid. MSSA was detected in the puncture fluid culture. On day 14, the blood culture showed no bacterial growth. He was treated with a sulfamethoxazole/trimethoprim (ST) mixture for the following six weeks and was discharged to his home with full activity of daily life.

## Discussion

This case report shows the difficulty in diagnosing prostatic abscesses in an immunocompromised host. Patients with immunocompromised conditions may not show typical symptoms such as advanced diabetes mellitus, as in this case. Bloodstream infection of MSSA can be critical, but immunocompromised hosts may show vague symptoms, such as fatigue and arthralgia [3]. Patients without hospital visits or annual health checks can have various undiagnosed diseases. General physicians should consider such patients comprehensively.

MSSA bacteremia should not be missed in patients with vague symptoms. The 90-day mortality rate from *S. aureus *bacteremia is as high as 29% [3,4]. MRSA bacteremia has an even higher risk of mortality [5,6]. Several observational studies have shown that vancomycin, a single agent for MSSA bacteremia, has a worse prognosis than β-lactam antibacterial agents [7-9]. The first step in treating prostatic abscesses is administering antimicrobials, and drainage should be performed if there is no improvement. In our case, we consulted a urologist who performed puncture drainage of the prostatic abscess on day 13 of hospitalization. *Staphylococcus aureus *rarely invades the urinary tract ascending into the urinary tract and causing urinary tract infection; rather, bacteremia causes renal and prostatic abscesses, and *S. aureus* is often detected in the urine [10]. In the present case, MSSA bacteremia was thought to have developed due to an abrasion on the left thigh after a fall one week before the visit, and the duration of treatment for bacteremia caused by *S. aureus *was determined by whether the patient had complicated bacteremia [11,12]. A minimum treatment period of 4-6 weeks after negative blood cultures is recommended for complicated bacteremia. Our patient’s case was complicated by persistent bacteremia; therefore, the treatment period was six weeks from when the blood culture became negative.

Unexplained systemic joint pain can indicate occult bacteremia. Our patient experienced pain in both upper extremities and elevated levels of inflammatory markers. The elevated neutrophil predominance in the leukocyte fraction indicates an infectious disease. Systemic inflammation caused by autoimmunity or infections can cause systemic pain in joints and muscles [13]. In our patient, MSSA bloodstream infection caused severe inflammatory conditions, which can trigger systemic joint pain. Systemic pain can appear in older patients and is treated as a medically unexplained symptom. For the effective diagnosis of occult bacteremia, blood culture and inflammatory conditions should be checked in older patients.

To overcome the difficulty in diagnosing occult bacteremia caused by *Staphylococcus*, general physicians who commonly approach multiple symptoms among older and immunocompromised patients should consider bacteremia in every encounter with such patients. Within the specialty of general physicians, system-specific approaches can be effective for older patients [14]. Considering multiple organ systems comprehensively requires precise history taking and physical examination, including vital signs [15]. Instead of focusing on one organ, a systematic analysis of symptoms can clarify the conditions of systemic inflammation caused by infections, including bacteremia [14]. Furthermore, immunocompromised hosts may exhibit vague and multiple symptoms without specific physical findings. To diagnose and treat them, family physicians should order blood cultures and look for infections in deep parts of the body, as in this case, which can clarify the effective management of older immunocompromised patients [16].

## Conclusions

This case involved a 72-year-old male with independent activities of daily living who developed prostate and perifemoral abscesses with multiple vague symptoms due to diabetes-related MSSA bacteremia. It is important to comprehensively evaluate multiple vague symptoms considering the immunological conditions of patients and investigate their suspicion of bacteremia and abscess in the deep parts of the body. Family physicians in rural contexts should deal with multiple symptoms among older immunocompromised patients.
